# Conversion Disorder: The Brain’s Way of Dealing with Psychological Conflicts. Case Report of a Patient with Non-epileptic Seizures

**DOI:** 10.7759/cureus.3902

**Published:** 2019-01-16

**Authors:** Delaram Pourkalbassi, Pooja Patel, Patricio S Espinosa

**Affiliations:** 1 Neurology, Marcus Neuroscience Institute at Boca Raton Regional Hospital, Boca Raton, USA

**Keywords:** conversion disorder, functional neurological symptom disorder, psychogenic non-epileptic seizures, todd’s paralysis, psychological conflicts

## Abstract

Conversion disorder or a functional neurological symptom disorder is a psychiatric illness in which psychological conflicts are manifested as physical symptoms. Common examples of symptoms include blindness, paralysis, dystonia, anesthesia, inability to speak, difficulty swallowing, incontinence, balance problems, tremors, difficulty walking, hallucinations, and psychogenic non-epileptic seizures (PNES). Conversion disorder is often missed on initial medical and neurological evaluations due to the lack of a definitive organic diagnosis. This case highlights the presentation and diagnostic complication of a patient with conversion disorder and emphasizes the importance of implementing a multidisciplinary approach to the treatment of this disorder, including clinician-patient proper communication, proper neurological/epilepsy evaluation, psychiatric therapy, psychotherapy, physical therapy, and pharmacotherapy.

## Introduction

Conversion disorder or functional neurological disorder (FND) is a psychiatric condition in which the body’s emotional and psychological stressors are converted to physical symptoms that cannot be explained by a neurological or medical condition. Psychogenic symptoms usually arise in response to stressful, traumatic events or psychiatric disorders. Common symptoms include blindness, paralysis, dystonia, anesthesia, inability to speak, difficulty swallowing, incontinence, balance problems, tremors, difficulty walking, hallucinations, and psychogenic non-epileptic seizures (PNES) [1-2]. It is important to differentiate conversion disorder from other somatoform disorders, such as factitious disorders and malingering, in which patients feign their symptoms.

Although conversion disorder symptoms are not caused by organic diseases, the symptoms are not intentional or under the conscious control of the patient [2]. Patients diagnosed with conversion disorder should seek immediate medical attention for comprehensive workup, as the symptoms may be associated with many other neurological and psychiatric disorders. Here, we describe a case of a patient with recurrent seizures and prolonged paralysis of the left side of the body.

## Case presentation

A 41-year-old male with a past medical history significant for a reported history of post-traumatic coma, post-traumatic epilepsy with prolonged (three to four days) Todd’s paralysis, bullet wounds to the head, two ocular strokes with left eye blindness, coronary artery disease (CAD), post-percutaneous coronary intervention (PCI), hypertension, benign prostatic hyperplasia (BPH), spinal stenosis, attention deficit hyperactivity disorder (ADHD), and bipolar disorder presented to the hospital with reported multiple seizures and left-sided post-ictal paralysis. The patient had a history of multiple admissions to the hospital due to seizures. He stated that he began having seizures after being attacked 24 years ago, where he sustained multiple injuries to the head, and claimed that he was in a coma for one year. Since then, he has carried the diagnosis of post-traumatic epilepsy for over 20 years. He reports having eight to nine seizures per month with associated “Todd’s paralysis,” which, according to him, resolves on its own after three to four days. He has been seen by multiple neurologists and has tried multiple antiepileptic drugs without seizure control. During the present admission, the patient was on phenytoin 400 mg and levetiracetam 1000 mg. He had therapeutic levels of both medications during this admission.

On examination, the patient’s mental status and cranial nerves examination were normal; the motor exam was significant for paralysis; and strength was 0/5 in the left arm and leg. He also complained of hemi-sensory loss on the left side of the body that was significant for no reaction to noxious stimuli. Reflexes were symmetrical and 2+ bilaterally. The patient was not able to ambulate due to the weakness. The psychiatric examination was significant for anxiety and auditory hallucinations. During an interview, the patient was slightly guarded, irritable, and talkative but redirectable.

**Figure 1:**
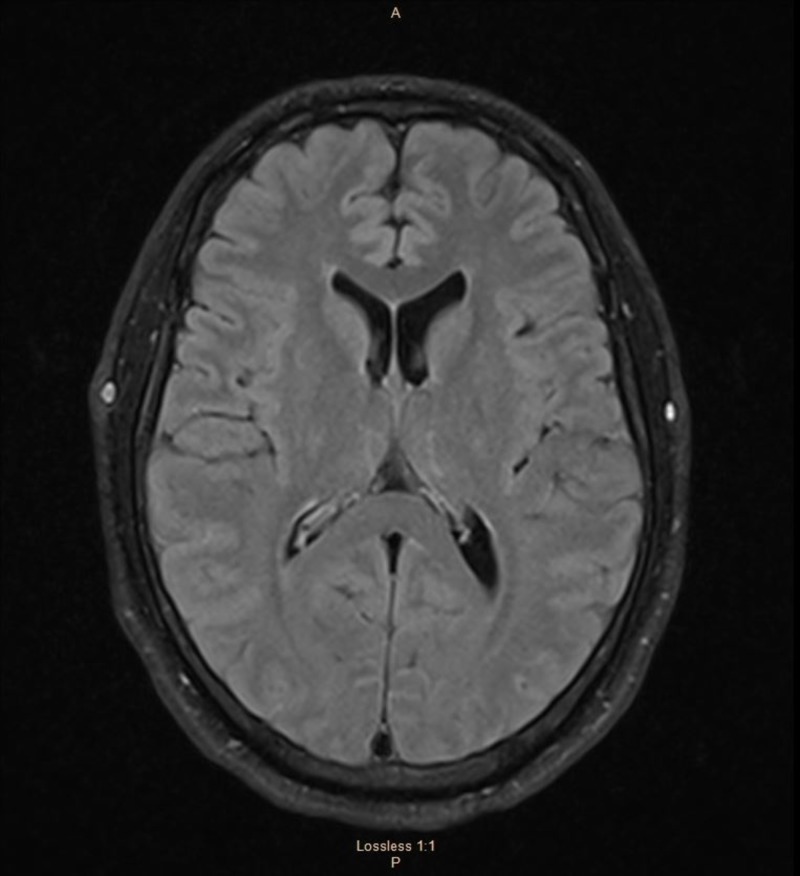
MRI of the brain FLAIR sequence; axial images within normal limits There is no evidence of traumatic brain injury or bullet wound injury, as reported by the patient. MRI: magnetic resonance imaging; FLAIR: fluid-attenuated inversion recovery

**Figure 2:**
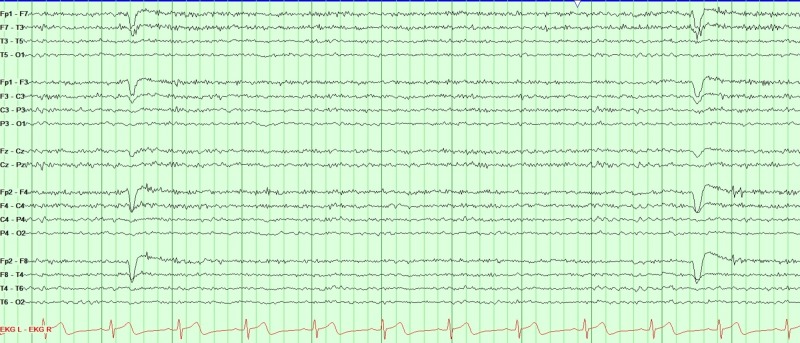
Electroencephalogram double banana montage Electroencephalogram (EEG) shows a normal background. There is no evidence of asymmetry or abnormality in relation with the patient's complains of functional post-ictal left-sided weakness.

Magnetic resonance imaging (MRI) of the brain was normal; specifically, there was no evidence of traumatic brain injury or any bullet injury as reported to and by the patient (Figure 1). Routine electroencephalography (EEG) was normal (Figure 2). Due to the lack of definite evidence of epilepsy, a video-EEG with medication titration was performed continuously for five days. The study was normal, with no focal or generalized epileptiform abnormalities noted. During the recording, there was no EEG correlation with the patient’s complaints of left-sided weakness. The EEG background was normal (typically, a patient with such severe weakness should have a slowing in the right hemispheric region). On the first day of the recording, when he had reported left-sided weakness, he was noted to have movements on the left side during sleep. Given that there was no evidence of epilepsy and psychogenic weakness, the patient was successfully weaned off all anti-seizure medications.

The patient was discharged home after six days fully functional: walking, speaking, and eating on his own. He was glad to know that he did not have epilepsy and was very thankful for the care and diagnosis received.

## Discussion

This case report shows many of the important characteristics of conversion disorder, such as the manifestation of a somatic deficit after psychological distress that is not better explained by another medical or mental disorder, lack of a definitive organic diagnosis, prolonged psychogenic paralysis, and episodes of psychogenic non-epileptic seizures, resembling epileptic seizures (Table 1). Psychoanalytic theory postulates that conversion disorder is caused by the repression of unconscious intrapsychic conflicts and conversion of anxiety into physical symptoms [2]. Unlike epileptic seizures, psychogenic non-epileptic seizures are not a result of an organic brain disease. Instead, they are stress-induced and result from traumatic psychological experiences, at times from a forgotten past. Such a disorder can also be viewed as a physical communication disorder, where distress is expressed somatically, rather than in a healthy verbal manner [3].

**Table 1:** Clinical presentation and distinguishing features of conversion disorder

Symptom	Conversion/Psychogenic	Organic
Weakness/Paralysis (Hemiparesis)	Initial strength followed by a "give-way quality” [4] Symmetric tone and reflexes including DTRs [4] Usually no difference between extensor vs flexor [4] Hoover’s sign present: Lack of reflexive involuntary contralateral hip extension [4] Negative pronator drift test: lowering of weak limb, without pronation [4]	Weakness Usually asymmetric tone and reflexes [4] Distal to proximal gradient [4] Hoover’s sign absent: Reflexive involuntary contralateral hip extension [4] Positive pronator drift test: pronation of weak limb [4]
Gait Disturbance	Inconsistent presentation with distraction of the patient [4] Bizarre gait patterns, especially tandem walking with excessive arm waving and body swaying [4] Drags paretic leg [4]	Consistent presentation and pattern [4] Circumducts leg [4]
Tremor	Increase of tremor amplitude when more weights are added to the affected limb [1]	Decrease of tremor amplitude when more weights are added to the affected limb [1]
Anesthesia	The sensory disturbance is not in a cortical, dermatomal or peripheral nerve distribution [4] Reduced activation in somatosensory cortices [5] May occur anywhere but most common on the extremities [1] Areas of anesthesia have a very precise and sharp boundary, often located at a joint [1]	The sensory disturbance is in a cortical, dermatomal or peripheral nerve distribution and consistent from examination to examination [4]
Blindness	No expected bruises or scrapes [1] Pupillary reflex present Intactness of the optic nerve, chiasm, tract, lateral geniculate body, and mesencephalon [1] Intermittent blurred vision, double vision nystagmus, visual field defects, and complete blindness [5] Reduced activation in the visual cortex [5]	Usually bruises or scrapes due to physical injuries Pupillary reflex absent Injury to the visual pathways. i.e. Stroke, Optic neuritis, tumor, etc.

The diagnosis of conversion disorder can be challenging since computed tomography (CT) and MRI of the brain and EEG typically show no abnormalities. However, long-term monitoring, such as inpatient video-EEG monitoring and ambulatory video-EEG recording in addition to a thorough psychiatric history and physical examination findings offer reliable diagnostic tools with high levels of certainty [1,3]. A review of the mental status and neurologic and physical examination results of the presented case raised clinical suspicions for conversion disorder. 

In our case, the patient had an event of psychogenic paralysis with no EEG correlate and had a weakness though no unilateral hemispheric slowing was noted. Normal EEG during these times suggested a non-organic cause. In addition, the fact that Todd’s paralysis does not typically last longer than 24-48 hours was another piece of evidence that helped establish the correct diagnosis. The Diagnostic and Statistical Manual of Mental Disorders, fifth edition (DSM-5) [2] diagnostic criteria for conversion disorder can assist clinicians in diagnosing conversion disorder after ruling out organic causes. Table 2 helps summarize useful tools in differentiating psychogenic non-epileptic seizures from epileptic seizures.

**Table 2:** Main differences between pseudoseizures and epileptic seizures

Pseudoseizure (Psychogenic Non-epileptic)	Epileptic Seizure
Less stereotyped (inconsistent appearance from one episode to another) [4]	Usually stereotyped [4]
Emotional trigger common [4]	Variable or no specific trigger [4]
Gradual evolution [4]	Quick evolution [4]
Longer duration [4]	Usually seconds to minutes [4]
Usually maintained consciousness [4]	Usually impaired consciousness [4]
Tongue biting, urination and injury rare [4]	Tongue biting, urination and injury common [4]
Limb moves away from face (when plegic arm is placed above face) [4]	Limb hits face (when plegic arm is placed above face) [4]
Resists eyelid opening and may present with visual fixation [4]	No resistance to eyelid opening and spontaneous eye movements [3]
Retained ability to follow commands or talk during an event [4]	May not completely impair an ability to follow commands or talk [4]
Frequent psychiatric history [4]	Uncommon psychiatric history [4]
No electrographic seizure activity in video-EEG [4]	Usually electrographic seizure activity in video-EEG [4]

This case also exemplifies the importance of prolonged video-EEG monitoring and the expertise that neurologists with epilepsy training can bring for optimal management and a successful treatment program [3]. By analyzing the video-EEG study, the diagnosis can be made with certainty by experienced epileptologists. One of the vital approaches for the treatment of patients with conversion disorder is the neurologist’s tactful presentation of the diagnosis [2-3]. Many patients who experience symptoms related to conversion disorder are unable to understand this inner conflict, which is perhaps occurring at a subconscious level [2]. It is important to educate patients to understand the presence of the psychological underpinnings but, more importantly, patients should be made aware of the connection between the conflict and the physical symptoms, and this should be communicated to the patient through an empathetic and unambiguous approach [3]. Once the patient recognizes this connection, they are more likely to accept the diagnosis and respond to appropriate treatment [2].

Furthermore, psychotherapeutic approaches, including individual or group therapy, behavioral therapy, hypnosis, biofeedback, and relaxation training, are reported to be the most-effective treatment methods [3]. To date, among all psychotherapy methods, cognitive-behavioral therapy has shown the highest level of efficacy for the treatment of patients with PNES.

For patients with motor symptoms, physical therapy can improve the physical symptoms and prevent secondary complications; however, the most important prognostic factor is the acceptance and understanding of diagnosis. Another effective treatment method is the use of antidepressants, anxiolytic, and other psychiatric medications, which is necessary for the treatment of underlying psychiatric problems [1]. For the case presented here, treatment ultimately involved reassuring the patient and family that there was no underlying neurological condition and no evidence of epilepsy and emphasizing and helping them understand the diagnosis of conversion disorder.

## Conclusions

Psychogenic non-epileptic seizures fall into the category of conversion disorder. Conversion disorder, part of somatoform disorders, is a psychiatric condition in which psychological conflicts are manifested as physical symptoms. Patients with conversion disorder present a diagnostic challenge due to their complex presentation. A multidisciplinary approach to the treatment of conversion disorder, including the clinician-patient relationship, and proper communication, correct neurological/epilepsy evaluation, diagnosis, treatment, psychiatric therapy, psychotherapy, physical therapy when needed, and pharmacotherapy provide the most promising results.
